# Supplementary material for: In vitro interactions of berbamine hydrochloride and azoles against Aspergillus fumigatus
Source: Microbiol Spectr. 2025 Mar 31;13(5):e03184-24. doi: 10.1128/spectrum.03184-24 (PMC12054048; doi:10.1128/spectrum.03184-24)
Supplement: Supplemental material — (i) Generation of deletion mutants. (ii) Strain identification. (iii) Gene expression analysis. Figure S1: Schematic diagram of the chemical structure of berbamine hydrochloride. Figure S2: Schematic overview of fusion PCR-based generation of gene knockout mutants in A. fumigatus. Table S1: Information on missing genes in gene-deficient strains. Table S2: Primer sets and corresponding amplification targets. Table S3: Real-time quantitative PCR primer list. Table S4: Primer list of generation of deletion mutants. [file spectrum.03184-24-s0001.docx]

**1. Generation of deletion mutants**

*A. fumigatus* AF293 (Fungal Genetics Stock Center), used as the parent strain for all amplification in this investigation, includes the whole DNA sequence of *A. fumigatus*. *A. fumigatus* A1160 (ΔKU80, *pyrG^-^*) (Fungal Genetics Stock Center) is a uracil (U)-deficient strain, defective in the *pyrG* gene, and unable to grow on U-deficient media.

1.1 Preparation of Knockout Product: The target gene sequence was retrieved from the NCBI database, and primers P1+P2 and P3+P4 were designed for the target gene. Genomic DNA of AF293 was used as a template to amplify 1200 bp sequences upstream and downstream of the target gene, obtaining the flanking regions. The plasmid pBARGPE1-Pyrg-TagRFP (Wensheng Biotechnology, Hunan, China) DNA was extracted as the amplification template, and primers PyrG-F and PyrG-R were used to amplify the *pyrG* gene sequence, obtaining the *pyrG* fragment. Finally, using primers P5+P6, along with the upstream and downstream fragments and the *pyrG* fragment, PCR amplification was conducted to obtain the complete knockout product (Fig.S1,Table S4).

1.2 Preparation of Protoplasts: 150 μL of A1160 spores (1×10⁹ cfu/mL) were inoculated into 50 mL of SAB liquid medium containing uracil (0.05 g/100 mL) in a sterile 50 mL flask, which was sealed with sterile foam and covered with sterile aluminum foil. The flask was incubated at 37°C and 130 rpm for 16 hours. After spore growth, mycelium was collected using miracloth (Millipore Sigma) and transferred to a new sterile 250 mL conical flask. Pre-prepared protoplast solution[1] was added, and the mixture was incubated on a shaking incubator at 37°C, 100 rpm/min for 4 to 5 hours until most spores had transformed into protoplasts. Miracloth was used to remove incompletely digested mycelium.

1.3 The filtered solution was transferred to a 50 mL conical tube and centrifuged (1800 g, 4°C, 10 minutes). After removing the supernatant, the pellet was resuspended in 2 mL of KCl/CaCl₂ solution and divided into two sterile 1.5 mL EP tubes. The suspension was further centrifuged at 4°C, 900 g for 3 minutes, and the supernatant discarded. This process was repeated twice, and the protoplasts from both tubes were combined into one tube. A final centrifugation (1800 g, 4°C, 3 minutes) was performed, and the supernatant was discarded. A 1 μL aliquot of the suspension was placed on a hemocytometer, and the concentration was determined using a microscope. The final concentration was adjusted to 1×10⁷ cfu/mL, and the final protoplast solution was prepared by mixing with 1 mL of KCl/CaCl₂ solution.

1.4 Transformation Procedure: 20 μL of the fusion PCR product was added to a sterile EP tube, followed by 20 μL of filtered PEG solution and 50 μL of the prepared protoplast suspension. The mixture was gently pipetted and incubated on ice for 30 minutes. An additional 100 μL of filtered PEG solution was added, and the mixture was pipetted again and incubated on ice for 5 minutes to allow the fusion product, which carries the marker gene, to successfully enter the protoplasts and undergo homologous recombination.

1.5 The mixture was plated onto CZA solid medium without uracil and incubated at room temperature for 24 hours. Afterward, the plate was transferred to a 37°C incubator and cultured for 3 to 5 days, and transformants were selected.

**2. Strain identification**

The cultured fungal specimens were taken and preliminarily identified according to the morphologic characteristics. Fungal DNA was extracted by MolPure Fungal DNA Kit and further amplified the ribosomal DNA transcriptional spacer internal transcribed spacer (ITS), beta-tubulin and calmodulin genes (Table S2)[2-5], PCR was performed using the following parameters: 3 min at 95℃, followed by 35 steps of 1 min at 95℃, 1 min at 58.5 ℃ and 1 min at 72 ℃, and then a final 10 min at 72 ℃. The final products were sequenced by Biocompany [BioEngineering (Shanghai) Co., Ltd], and finally, the sequence was blasted in NCBI GenBank. The definitive identification of the *Aspergillus* isolates was accomplished by comparing the sequences with relevant reference sequences in GenBank using the nucleotide BLAST system (https://blast.ncbi.nlm.nih.gov/Blast.cgi).

**3.Gene expression analysis**

*A. fumigatus* conidia were collected after 48 hours of incubation on SAB solid medium at 35℃ and resuspended in 1640 liquid medium to a final concentration of 5×10⁴ cfu/mL. All samples were incubated at 37℃ in a shaking incubator at 130 rpm for 16 hours. During the incubation period, drug treatments were applied as needed. Total RNA was extracted using TRIeasy (Yeasen Biotechnology, Shanghai, China) and reverse-transcripted into cDNA using a Hifair® Ⅲ 1st Strand cDNA Synthesis SuperMix (Yeasen Biotechnology, Shanghai, China). Concentrations and quality were also determined using Nanodrop one (ThermoFisher Scientific). PCR was performed using the following parameters: 5 min at 25℃, 15 min at 55℃ and 5 min at 85℃. RT-qPCR was performed in triplicate on Real-Time Quantitative Thermal Cycler (MA-6000, Yarui Biotechnology, China) with Hieff qPCR SYBR Green Master Mix (Yeasen Biotechnology, Shanghai, China).The relative gene expression level (2^-ΔΔCT^) was calculated using the actin housekeeping gene control[6]. All primers used for gene expression analysis are listed in [Supplemental Table S3](https://pmc.ncbi.nlm.nih.gov/articles/PMC9516190/#sup1).


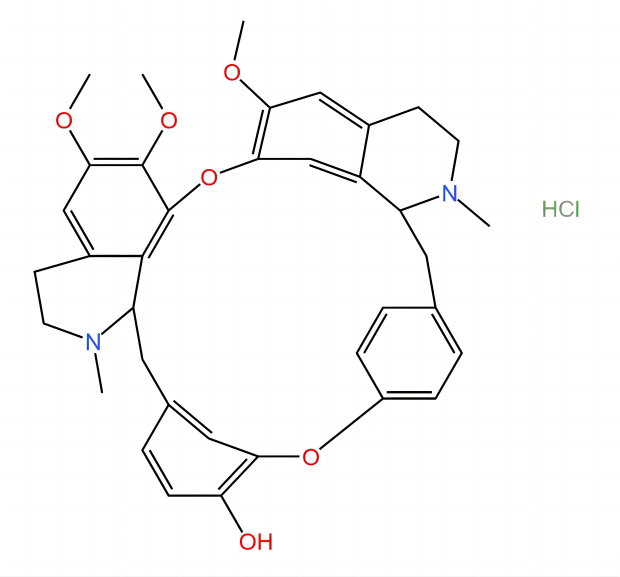


**Fig.S1** Schematic diagram of the chemical structure of berbamine hydrochloride

Note: Berbamin hydrochloride is a bioactive alkaloid with an isoquinoline ring at its core, which provides structural stability and affects its physical and chemical properties. The molecule has multiple methoxy groups (-OCH3), which plays an important role in biological activity and solubility.


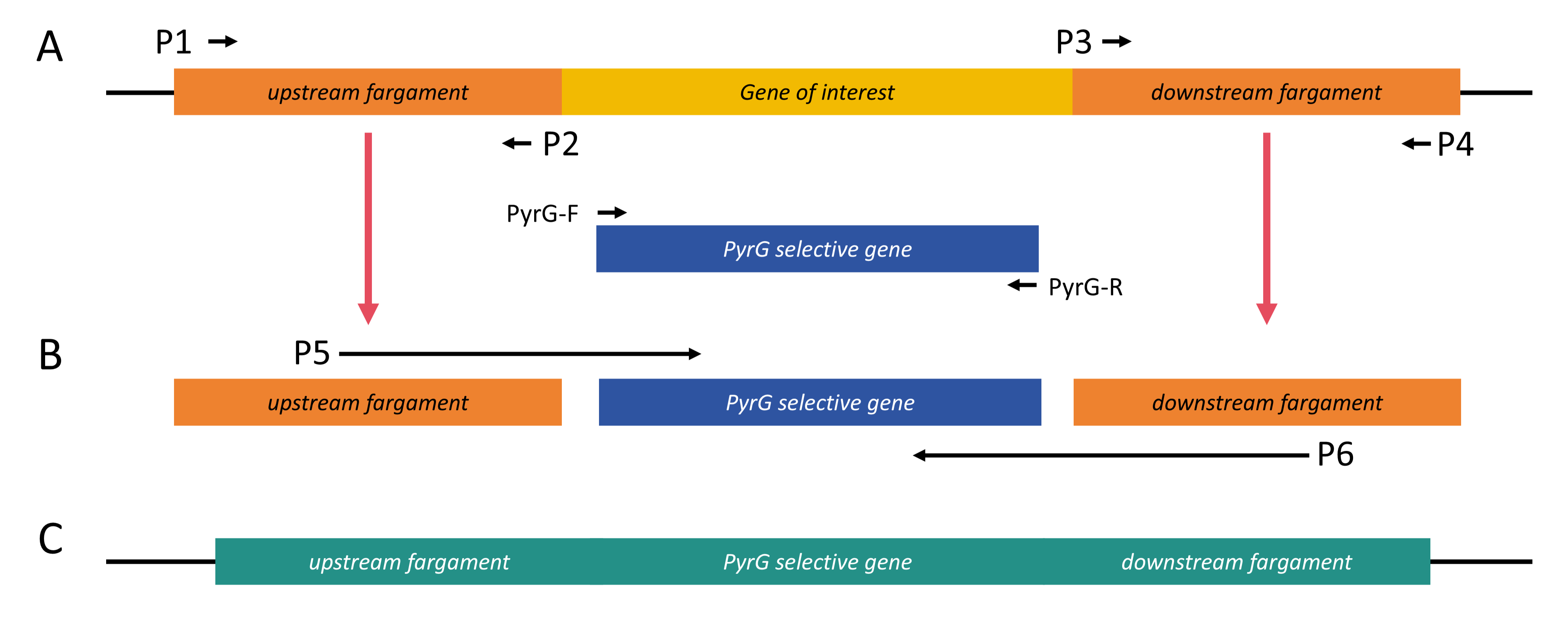


**Fig.S2** Schematic overview of fusion PCR based generation of gene knockout mutants in *A. fumigatus*

Note: **(A)** Upstream and downstream fragments are amplified with primers P1 and P2 and with P3 and P4. The *pyrG* selective marker cassette is amplified with primers PyrG-F and PyrG-R. **(B)** The upstream and downstream fragments are fused to the*pyrG* selective cassette by fusion PCR using nested primers (P5 and P6) creating a linear fragment suitable for transformation.**(C)** The final knockout product generated.

**Table S1.** Information on missing genes in gene-deficient strains

| Name | Code | Annotation |
| --- | --- | --- |
| △*mfs01* | AFUA_1G03200 | MFS transporter, putative |
| △*mfs02* | AFUA_1G05010 | MFS multidrug transporter, putative |
| △*mfs03* | *AFUA_1G12620* | *MFS toxin efflux pump (AflT), putative* |
| △*mfs04* | AFUA_1G16910 | MFS multidrug transporter, putative |
| △*mfs05* | AFUA_2G05840 | MFS multidrug transporter, putative |
| △*mfs06* | AFUA_2G11420 | MFS transporter, putative |
| △*mfs07* | AFUA_3G02060 | MFS multidrug transporter, putative |
| △*mfs08* | AFUA_3G02720 | MFS transporter, putative |
| △*mfs09* | AFUA_3G03190 | MFS multidrug transporter, putative |
| △*mfs1*0 | AFUA_3G08530 | MFS drug transporter, putative |
| △*mfs11* | AFUA_3G13520 | MFS drug transporter, putative |
| △*mfs12* | AFUA_4G03920 | MFS drug transporter, putative |
| △*mfs13* | AFUA_5G01350 | MFS multidrug transporter, putative |
| △*mfs14* | AFUA_5G01540 | MFS aflatoxin efflux pump, putative |
| △*mfs15* | AFUA_5G14490 | MFS transporter, putative |
| △*mfs16* | AFUA_6G00710 | MFS transporter, putative |
| △*mfs17* | AFUA_6G01860 | MFS lactose permease, putative |
| △*mfs18* | AFUA_6G01930 | MFS transporter Seo1, putative |
| *△mfs19* | AFUA_6G02220 | MFS toxin efflux pump, putative |
| △*mfs20* | AFUA_8G04150 | MFS toxin efflux pump, putative |
| △*abc1* | AFUA 2G15130 | ABC multidrug transporter, putative |
| △*abc2* | AFUA_3G09680 | ABC efflux transporter, putative |
| △*abc3* | AFUA_4G09150 | ABC multidrug transporter, putative |
| △*abc4* | AFUA_5G09480 | ABC transporter, putative |
| △*cdr1B* | AFUA_1G14330 | [ABC transporter, putative](https://www.ncbi.nlm.nih.gov/gene/3509814) |
| △*tca1* | AFUA_3G07810 | succinate dehydrogenase subunit Sdh1, putative |
| △*tca2* | AFUA_5G14880 | [mitochondria protein Fmp29, putative](https://www.ncbi.nlm.nih.gov/gene/3510952) |
| △*tca3* | AFUA_6G03590 | citrate synthase Cit1, putative |
| △*tca4* | AFUA_1G12800 | isocitrate dehydrogenase, NAD-dependent |
| △*tca5* | AFUA_6G07390 | isocitrate dehydrogenase LysB |
| △*tca6* | AFUA_5G14880 | mitochondria protein Fmp29, putative |

**Table S2.** Primer sets and corresponding amplification targets

| Target gene | Primer | Primer DNA sequence(5’-3’) |
| --- | --- | --- |
| ITS | ITS1 | TCCGTAGGTGAACCTGCGG |
|  | ITS4 | TCCTCCGCTTATTGATATGC |
| calmodulin | cmd5 | CCGAGTACAAGGAGGCCTTC |
|  | cmd6 | CCGATAGAGGTCATAACGTGG |
| beta-tubulin | Bt2a | GGTAACCAAATCGGTGCTGCTTTC |
|  | Bt2b | ACCCTCAGTGTAGTGACCCTTGGC |

**Table S3.** Real-time quantitative PCR primer list

| primer | sequence(5’-3’) |
| --- | --- |
| cdr1B qPCR-F | GAGTGCGTACGATGTATTCGAC |
| cdr1B qPCR-R | GGCAGGACTGGTGAGAGAAG |
| Actin-F | GCACGTGAAATTGTTGAAAGG |
| Actin-R | CAGGCTGGCCGCATTG |

**Table S4.** primer list of Generation of deletion mutants

| primer | | sequence(5’-3’) |
| --- | --- | --- |
| PyrG-F | | CCGGCTCGGTAACAGAACTACCGCAGACAATGCTCTCTATC |
| PyrG-R | | GTTGGAGCATATCGTTCAGAGCAATACCGTTACACATTTCCA |
| △*mfs01* | P1 | ACTATGAGTGATAGGAGACATTGG |
|  | P2 | TAGTTCTGTTACCGAGCCGGCCTAGCCACCTCCTGAACAGA |
|  | P3 | GCTCTGAACGATATGCTCCAACTCCGTTACAAGACGTGCCCTG |
|  | P4 | GTATGACTGTAATCGCCGGCA |
|  | P5 | GCGACAATCAGTGTTCTTTGAAAC |
|  | P6 | GCAGATGTATACAGGGGCAGCG |
| △*mfs02* | P1 | TCAATAACGTGGATGTGACCTCG |
|  | P2 | TAGTTCTGTTACCGAGCCGGCGGAATGCAAGCGCTGTCA |
|  | P3 | GCTCTGAACGATATGCTCCAACCAAGCATAGAGCAGCACACAT |
|  | P4 | CATAAGTGGCAGGATCCGACCTAT |
|  | P5 | GGAAGCCTATCTTCAGTGTTG |
|  | P6 | TGAAAGCAACAGGTCTGTGC |
| △*mfs03* | P1 | GCTTGGATGGCTGAAAATCCGG |
|  | P2 | TAGTTCTGTTACCGAGCCGGTTTGACTCGCTTATGTCGGGG |
|  | P3 | GCTCTGAACGATATGCTCCAACGTGGTCATCCACAGACTTTTCTTTT |
|  | P4 | GCCAAGGGAGATGATCCACTT |
|  | P5 | TTCCTACTAGTCTAGCGGTAATTAG |
|  | P6 | ACTCGTCCTGATCGATTAGGAC |
| △*mfs04* | P1 | ACCACTCGTCTATGCCTTTTTCC |
|  | P2 | TAGTTCTGTTACCGAGCCGGTGTACGAAACCCCGAGCG |
|  | P3 | GCTCTGAACGATATGCTCCAACAGTTCTATCCTTGCGAATTTTCTCT |
|  | P4 | GCCTTTGCTCAACTTCACCG |
|  | P5 | TAGTATCTCCGAGTTGCCTACGCA |
|  | P6 | GGCAATCGGGTTCTCTACCC |
| △*mfs05* | P1 | TTGCCAATTCTCCTTTCTGGCT |
|  | P2 | TAGTTCTGTTACCGAGCCGGCCTTATTCACATAGGTCGAATCGA |
|  | P3 | GCTCTGAACGATATGCTCCAACCCTGTTGTTACGAACTGCGATG |
|  | P4 | CCATCAGCTAACACCAACCTCAAC |
|  | P5 | CATCCAGAGCTGCAATTGCTA |
|  | P6 | CCCAGTTTCGGGAAATGCCT |
| △*mfs06* | P1 | TGTGGCGGGAGTTATCTGAAAG |
|  | P2 | TAGTTCTGTTACCGAGCCGGTTAGGTTGCCATGCTGGTGAAG |
|  | P3 | GCTCTGAACGATATGCTCCAACCGGTTGATTCCCTTCTCCGAT |
|  | P4 | ACTAACTTCGAAACCAGATTCACTC |
|  | P5 | GGCTATTTCGTAAGGTTGATCGG |
|  | P6 | GAAGACCATTTCAACCGCCATC |
| △*mfs07* | P1 | GAACTGTGGGACAATCGCTCAT |
|  | P2 | TAGTTCTGTTACCGAGCCGGGGTTGGATTATAAAGGAAGCCCTCG |
|  | P3 | GCTCTGAACGATATGCTCCAACTTCATTATAATCCACGATAGACTAT |
|  | P4 | ACCATCTGTTCATATATTCTCCT |
|  | P5 | TTTTCTTTGGTGTCTACAGAAGCGG |
|  | P6 | GAGGATTGCTTATTCTATGCCCC |
| △*mfs08* | P1 | TCTAGAATATGGACTCGAGATCCAG |
|  | P2 | TAGTTCTGTTACCGAGCCGGTTTCTTTTTCACCGCTGTTTATAAA |
|  | P3 | AGAAGGCTGCACGTCATTAATCT |
|  | P4 | AGCCAAGAAGGTCAACCGC |
|  | P5 | TGAATAGCTTCAAGAGGCCAAGT |
|  | P6 | TACCAAGTCCTGTCTACCTCGT |
| △*mfs09* | P1 | GTGACATACGATCGTGATGTGTGT |
|  | P2 | TAGTTCTGTTACCGAGCCGGGATGTGCGACTGATGATAGACAG |
|  | P3 | GCTCTGAACGATATGCTCCAACGGATGCCAGCAGCTTTCATC |
|  | P4 | ACATCCGAACCCGATACATCATAT |
|  | P5 | CATCGGTATTTGGTCAGACTGTC |
|  | P6 | CTATAGTGTCGCTGCATTCTTTCC |
| △*mfs10* | P1 | CTCTATACCACGTTTGAGAGCAAG |
|  | P2 | TAGTTCTGTTACCGAGCCGGGATTTGAGCAGAATAGAACGAGTCG |
|  | P3 | GCTCTGAACGATATGCTCCAACCCCTCCTCCCTAGCTTCTTCATC |
|  | P4 | TCAGTCAGCTCCTGATATCAAGC |
|  | P5 | GCTTCGAGGCAGTCTGGTC |
|  | P6 | TCTGCCGGAGCAATTCTGAG |
| △*mfs11* | P1 | TGAGTTCTCTGGCTTCAGAGTCA |
|  | P2 | TAGTTCTGTTACCGAGCCGGTATCTACGTTGGTGTTACATGAGGG |
|  | P3 | GCTCTGAACGATATGCTCCAACGTTGTGTGGGAGGGTTGGATG |
|  | P4 | GATTTCAGGTTAAGTGTCGGTGA |
|  | P5 | CCACGGTGGTAGTCAGAAATACA |
|  | P6 | ACCGATGAGGTAATTTGGTCGC |
| △*mfs12* | P1 | GGCGATCATCGACATGGAGATTAC |
|  | P2 | TAGTTCTGTTACCGAGCCGGCGTGCTTTGCTAGTTGCTACGT |
|  | P3 | GCTCTGAACGATATGCTCCAACCTGTCGGTCTCGGTTAATTGCAG |
|  | P4 | GTTTACCATCCTTCCTCTAAAGGGA |
|  | P5 | CGGATGCATCGACATCATTTTGG |
|  | P6 | AAGAATTCGCTTCTCAACCTGTG |
| △*mfs13* | P1 | TATTAGCAGTCCGGTGGAATGAAG |
|  | P2 | TAGTTCTGTTACCGAGCCGGTCTCCGTCAGAAAGCTGGTCC |
|  | P3 | GCTCTGAACGATATGCTCCAACTATTAGCAGTCCGGTGGAATGAAG |
|  | P4 | TCTCCGTCAGAAAGCTGGTCC |
|  | P5 | GGCAATGATTTGATATGTGCTGGG |
|  | P6 | GTGGGAGATCCATACTTTTCCG |
| △*mfs14* | P1 | CATTGTAGCGTTGGCTTGGG |
|  | P2 | TAGTTCTGTTACCGAGCCGGGACCGTGCAAGGTAAGTTTCTG |
|  | P3 | GCTCTGAACGATATGCTCCAACCACTACTGTCTAATGCCTTTTGAGA |
|  | P4 | ACCTCGGTGAACTTTGCTC |
|  | P5 | TGTAGGTTTGCGTGGAGTTGC |
|  | P6 | CATGGAGTTGTTCCGTAGGCA |
| △*mfs15* | P1 | TTCTTAGCCAATGGCTTCTGGC |
|  | P2 | TAGTTCTGTTACCGAGCCGGATTGATCGTCACCTTGACTCTTATG |
|  | P3 | GCTCTGAACGATATGCTCCAACGAAAACAGTTCAGTGCATTTGTGTT |
|  | P4 | CCCAGGAGCAGTCTATCTATGCA |
|  | P5 | CAAGCGCTGCAGATGGAAATG |
|  | P6 | ATCCCGAGGAGATTCGGTACC |
| △*mfs16* | P1 | ATACGACGGGATTCCAAAGGC |
|  | P2 | TAGTTCTGTTACCGAGCCGGTGTGGAGATGCGGCAACTG |
|  | P3 | GCTCTGAACGATATGCTCCAACTGCAGTGAGGTTCGTAAAAGTAGGT |
|  | P4 | GGGATTAGAAGCTGAGAACTCAACC |
|  | P5 | CCGGTCAATCTCTTGTTGCCA |
|  | P6 | AAGCTTTCCTGATGCGGGTT |
| △*mfs17* | P1 | TCCATTACGCCGCAGGAGA |
|  | P2 | TAGTTCTGTTACCGAGCCGGGGTTTCCGCCTGAGACGAC |
|  | P3 | GCTCTGAACGATATGCTCCAACATGGTCGTCGGACTTATACAAATGG |
|  | P4 | TCGTTGTACACGACCCTGCT |
|  | P5 | TTGAGACATTGGGCCTGGTAC |
|  | P6 | TCTATCCAAATCCTGGCCGTAG |
| △*mfs18* | P1 | ATGACGATCTGGGTTGCTGTG |
|  | P2 | TAGTTCTGTTACCGAGCCGGTGCAGGAAGTCTCCGTTAGT |
|  | P3 | GCTCTGAACGATATGCTCCAACTGGAACTCTACTGAATGCAGGGG |
|  | P4 | GCTCATCATGCTCGATGGGAC |
|  | P5 | CATGGCCCTGGAAACACTC |
|  | P6 | CCTCCAAAGGTATCAGCCAGGA |
| *△mfs19* | P1 | TGCTGATGCCCCATTTATCATGTC |
|  | P2 | TAGTTCTGTTACCGAGCCGGGACGATGGGTAGGTGTTGGAGT |
|  | P3 | GCTCTGAACGATATGCTCCAACTTTTGTCTCTTAGATGACAGACTTC |
|  | P4 | CGGACAAGTCCAACTTATAAGG |
|  | P5 | GATCTGTCGCGGACTCTCG |
|  | P6 | GGGGAGAGAAAATCACAGTATGCT |
| △*mfs20* | P1 | TCTCATTTCTCAGAGACTAGCCAT |
|  | P2 | TAGTTCTGTTACCGAGCCGGAAGCCCCCAAACACTATGGC |
|  | P3 | GCTCTGAACGATATGCTCCAACAGTGAAGATACCATGTATGAGTG |
|  | P4 | ATTCCCTCGTTGCGTTTTTTCTC |
|  | P5 | TTAGTGCTGGCAAGGGACATT |
|  | P6 | TAGACTTGAATGTCTGGCATGTCAC |
| △*abc1* | P1 | GAGCTGAGTGACTGACTCCCA |
|  | P2 | TAGTTCTGTTACCGAGCCGGCGCTGCACGACTCACACC |
|  | P3 | GCTCTGAACGATATGCTCCAACGGGGAACGGACCTGACTTATTG |
|  | P4 | CAAATCACTCCCCCATGACTCG |
|  | P5 | ATCCAGCCATCCAGACAAGC |
|  | P6 | CCTTTGGGTTTTTCGTACTTTCTGG |
| △*abc2* | P1 | TTTCTTTTGATTGTATCCTGCCTGT |
|  | P2 | TAGTTCTGTTACCGAGCCGGGTCGAAACGGGCTTACTGCG |
|  | P3 | GCTCTGAACGATATGCTCCAACGGTAGACTTGACATTGTCTCTTGGT |
|  | P4 | TTGGCAACTAGACCGCTAGC |
|  | P5 | ATATATTAACCTCGTCCCTCGCAAT |
|  | P6 | ACACTGGGACATGACATTCTCT |
| △*abc3* | P1 | GAACTCAGATAAGGCTAACCTGGAA |
|  | P2 | TAGTTCTGTTACCGAGCCGGCTCTTTTTGTTTGTGAATCAGCCTG |
|  | P3 | GCTCTGAACGATATGCTCCAACTGTGATTGTTCGAGTATATCACCTC |
|  | P4 | CGTACGTTGTTGTTTGTTGTTG |
|  | P5 | TGGGGAAGAAATGTATCACCTCG |
|  | P6 | AAGCTCTTCAGAGATGCAGGG |
| △*abc4* | P1 | CTTTCCTTCTCCGAAATGAGCTCA |
|  | P2 | TAGTTCTGTTACCGAGCCGGGGTCGCCTGTAGTTCGATAAGTC |
|  | P3 | GCTCTGAACGATATGCTCCAACACCCGGACCTTGTATTAGCC |
|  | P4 | TGGTTCGAGCTGAGTATTTTCG |
|  | P5 | TGAGGTATGTACTAGCATCTGGTG |
|  | P6 | TCTTGCTTGCAAGTCAGCGA |
| △*cdr1B* | P1 | AATGCAAATTCAACTCGGAGCTTAC |
|  | P2 | TAGTTCTGTTACCGAGCCGGGATGGCCGATTGAGTGAGACAC |
|  | P3 | GCTCTGAACGATATGCTCCAACCAGCCTGACCTCCTTCCCT |
|  | P4 | ACATCGAGGGCCCTTACTCC |
|  | P5 | GCCCAGGACCTTCTTGAGTT |
|  | P6 | CAACCCAGCAGATCTACCTCAT |
| △*tca1* | P1 | GCTCGTTTCGATGATGGAGCTG |
|  | P2 | TAGTTCTGTTACCGAGCCGGTGTGGGCGGATCAAAAGAAAATAGA |
|  | P3 | GCTCTGAACGATATGCTCCAACATGCATTCCTCTGCACCTGAT |
|  | P4 | GTCATCTCTCAGATGACAGACGTCA |
|  | P5 | TGGTCGTATTGACCGCAAGATC |
|  | P6 | GAAGATAGCAACGAACGCACGAA |
| △*tca2* | P1 | AATGTCCGGGTTAAAGAACTCCG |
|  | P2 | TAGTTCTGTTACCGAGCCGGGTCGAAACTTGACGTAACGTTCG |
|  | P3 | GCTCTGAACGATATGCTCCAACTTATGGTGCGCATTGGCTTCAA |
|  | P4 | TTCTGGAAGGCTCTGCGGTT |
|  | P5 | CCAGACCTAATGCGAAACCAG |
|  | P6 | CTTAAGCACTTGGACTTCCCGA |
| △*tca3* | P1 | TATCATCGGTTTTGAGGAGGAGAGC |
|  | P2 | TAGTTCTGTTACCGAGCCGGTGTGATGGGATGCTATCAAGGGA |
|  | P3 | GCTCTGAACGATATGCTCCAACTTTCTCACGAAAAACTCGACTGAC |
|  | P4 | GATATGTCGATTCAAGGCAGCTG |
|  | P5 | GAGGGGTCTCCACTATCTCGC |
|  | P6 | ACCTCAAAACGTACACAGATAGCC |
| △*tca4* | P1 | AAAGCAAGAACCGGTTCCGTA |
|  | P2 | TAGTTCTGTTACCGAGCCGGTGTGATATGTCCCCGGATCAAC |
|  | P3 | GCTCTGAACGATATGCTCCAACGGCACTTGCTGCTGAAATGTACTAT |
|  | P4 | GCATGCACAAACACTTCCGTAT |
|  | P5 | CTCAGCAGGCCATTCCTCAG |
|  | P6 | CCCCGACGGATCAAAAGACTT |
| △*tca5* | P1 | CGGATCCGCGGCTTCAATTAA |
|  | P2 | TAGTTCTGTTACCGAGCCGGTGTGAGCTTCGGGTGTTGTG |
|  | P3 | GCTCTGAACGATATGCTCCAACATGATGGAGGCTGCGATGG |
|  | P4 | CGACTCAAGGTCACAGCAGG |
|  | P5 | GCCAAATGGCGTCATTATCCG |
|  | P6 | TCTCCCAAAACACACCCCTACTT |
| △*tca6* | P1 | AATGTCCGGGTTAAAGAACTCCG |
|  | P2 | TAGTTCTGTTACCGAGCCGGGAAACTTGACGTAACGTTCGTTCA |
|  | P3 | GCTCTGAACGATATGCTCCAACTTATGGTGCGCATTGGCTTCA |
|  | P4 | TTCTGGAAGGCTCTGCGGT |
|  | P5 | GAATGGATCGCATGCAGTGATC |
|  | P6 | CAGACTGTTGAATTTGCGCCAAA |

**References:**

1. Qureshi IA, Rao KV: **Sparse-fur (spf) mouse as a model of hyperammonemia: alterations in the neurotransmitter systems**. *ADV EXP MED BIOL* 1997, **420**:143-158.

2. Glass NL, Donaldson GC: **Development of primer sets designed for use with the PCR to amplify conserved genes from filamentous ascomycetes**. *APPL ENVIRON MICROB* 1995, **61**(4):1323-1330.

3. Gu X, Cheng X, Zhang J, She W: **Identification of the Fungal Community in Otomycosis by Internal Transcribed Spacer Sequencing**. *FRONT MICROBIOL* 2022, **13**:820423.

4. Gao L, Xia X, Gong X, Zhang H, Sun Y: **In vitro interactions of proton pump inhibitors and azoles against pathogenic fungi**. *FRONT CELL INFECT MI* 2024, **14**:1296151.

5. Hong SB, Go SJ, Shin HD, Frisvad JC, Samson RA: **Polyphasic taxonomy of *Aspergillus fumigatus* and related species**. *MYCOLOGIA* 2005, **97**(6):1316-1329.

6. Livak KJ, Schmittgen TD: **Analysis of relative gene expression data using real-time quantitative PCR and the 2(-Delta Delta C(T)) Method**. *METHODS* 2001, **25**(4):402-408.
